# The Economy of Health, or the Stream of Human Life from the Cradle to the Grave, &c.

**Published:** 1837-07-01

**Authors:** 


### The Economy of Health, or Stream of Human Life, from the Cradle to the Grave, &c. By *J. Johnson,* M.D. Second Edition, greatly enlarged. Feb. 1837.

As no review of this work can be inserted in this Journal, a few extracts, without comment, may be fairly allowed. The following is the only one we shall present at present.

" The most important feature of the Ninth Septenniad is, the Grand Climacteric—an epoch that has been regarded, in all ages, with something like mysterious awe, as the most critical in human life. Popular opinions of this kind are generally based on observation, however inaccurate, and are rarely the offspring of mere fancy, or a superstitious combination of numbers. *Nine times seven* forms a remarkable—indeed an appalling multiple, and very few can apply it to themselves, without feelings of the penseroso kind!

But the 'Grand Climacteric' is not merely a popular superstition; it has engaged the attention, and occupied the pen of a modern physician of great distinction. As the Essay was written some twenty years ago, it wants that development which Sir Henry Halford's further experience would have rendered more valuable. It is not my intention, however, to draw from any other source than the evidence of my own senses on this occasion.

The changes in the balance of the constitution which began to shew themselves rather unequivocally in the Eighth Septenniad, become but too conspicuous, as the age of 60 is touched, in a great majority of both sexes. The Ninth Septenniad is clearly the 'fifth age' of Shakespeare, typified by the 'Justice,' possessed of a portly corporation, 'with good capon lined'—

> 'With eyes severe and beard of formal cut,
> Full of wise saws and modern instances.'

Where corpulency does not obtain at this period, a contrary state not unfrequently commences. The fluids of the body diminish in quantity—the softer parts shrink—and the solid parts, as bones, cartilages, ligaments, &c. become more condensed than ever. The vessels conveying the blood from the heart to all parts of the body, begin to partially ossify, and are thus greatly weakened at the junction of the indurated with the elastic portions, rendering them liable to give way from distention or pressure. The cartilages of the ribs being turned into bone, the chest loses much of its expansive and contractile capabilities, and the breathing is less easy, especially when the body is in motion. The joints grow stiff, and the muscles get flaccid. All the senses become much more obtuse—and the various appetites greatly diminished—some of them being almost annihilated. By short-sighted man this diminution of enjoyment, in the exercise of the senses and appetites, is keenly deplored, though it is wisely ordained by the Omniscient Architect. Were the appetites to remain unimpaired, while the material fabric is necessarily, but gradually breaking down, the weakened organs would be overpowered—and sudden death, or painful maladies would be the consequence. This is sometimes the case, even as it is, when the appetites are stimulated by provocatives, and the tide of enjoyment swells beyond the channels which were destined to confine it.

About this period, too, the teeth, in a vast majority of people, become deficient in number, and very inadequate to the important function of mastication! —while digestion, already weakened, is thus greatly embarrassed, by the additional labour imposed on the stomach. All the internal organs growing more torpid, the secretions necessarily get more scanty. The skin itself becomes more dry, shrivelled, and wrinkled—the veins are enlarged and blue, slowly propelling the vital current towards the heart. In fine, every structure and function in the body shew clear and unequivocal marks of deterioration, gradually, but steadily increasing!

Nor do the intellectual faculties remain unaffected, though they do not always evince a strict correspondence with the failure of corporeal functions. Imagination, wit, and memory may flag; but judgment, understanding, and wisdom remain firm as a rock. Sixty years' experience indeed of human vicissitudes converts temerity into caution—sanguine hope into cool calculation—castles in the air into habitations a little (and but a little) more durable on earth—credulity into doubt—confidence into suspicion—prodigality into parsimony—and contempt of danger into timidity and love of life.

These and various other changes, moral and physical, are so gradual, that they cannot be measured by any standard of days, weeks, or months—scarcely indeed of years. But, whether from original defect in the organization, accidental injuries sustained in the journey, or, what is more common, from overworking of the living machine, it not unfrequently happens that, about the ninth Septenniad (sometimes sooner sometimes later), a marked alteration takes place in the rate of progression, or rather retrogression. In the course of a single year, nay of a few months, the physiognomy will present a singular and inauspicious look of deterioration. The character of expression in the countenance is changed—the features are pinched—the eye is lack-lustre—the strength is greatly diminished—the flesh wasted or bloated—the voice feeble—the gait unfirm—the appetite in abeyance—the thirst often troublesome—the spirits unaccountably depressed; and all this, without any tangible or visible disease, to explain the sudden declension of the various physical powers!

This is the CLIMACTERIC itself; but not the Climacteric DISEASE. The functions are greatly impaired; but no vital organ has, as yet, been affected in structure. The truth is, that the organs of daily supply are now inadequate to repair the daily waste—and the laws of vitality are no longer able to counteract the chemical laws of decomposition. The whole material fabric is therefore gradually crumbling down. But I believe that very few touch the final goal of existence in this way—at least I have seen no example of the kind. This general dilapidation—this universal decadence of functional power having obtained, for a longer or shorter period, some particular organ or class of organs, gives way in function or structure more than the others, and then we have the '*Climacteric disease.*' Thus the absorbents are frequently the first to fail in their office—the ancles swell—and effusions take place into the cavities of the brain, chest, or abdomen, with corresponding symptoms. If the effusion be in the head, we have drowsiness, loss of memory, thickness of speech, diminution of muscular power, partial paralysis—and finally, apoplexy of the watery kind.

If the effusion be in the chest, we have cough, embarrassed respiration, inability to lie low in bed, breathlessness in ascending stairs, &c. &c. If the effusion be in the abdomen, dropsy is the 'CLIMACTERIC DISEASE.' If the organs of digestion and nutrition be the first to give way (which is very often the case), then we have atrophy or general wasting of the body—ending in dropsical effusions.

But it not infrequently happens that the HEART itself is the organ on which the 'CLIMACTERIC DISEASE' falls. It becomes enlarged in size, softened in structure, thinned in its walls, and imperfect in its valves. The effects of this disease are far more conspicuous in the function of respiration than in that of the circulation. As at the time Sir Henry Halford wrote, we had not the means of distinguishing diseases of the heart, by the stethoscope, which we now have, so the 'CLIMACTERIC DISEASE' has probably been supposed to fall on the LUNGS

when the heart was the seat of disease.* In the course of a long experience I have met with few instances of this kind. In those cases where the lungs were *apparently* affected, the heart was the organ primarily and essentially diseased. Every experienced practitioner, indeed, is now well aware of this fact. At the period alluded to, asthma was generally considered an affection of the lungs alone: at present, it is known that, in nine cases out of ten, it is attributable to, or combined with, disease of the heart.

And here we have a most important subject to consider. In the *climacteric decline*, and before any one particular organ breaks up—when we have a great deterioration of several functions, without marked disease of any one structure, is there any chance of checking the progress of decay, or staving off, for a time at least, the *climacteric disease?* This question is not so easily solved, even by experience, as might be expected—and for this reason—that all the phenomena of the climacteric decline occasionally present themselves in people who are very far short of the ninth Septenniad—and where recovery often takes place. There is no reason why the same might not occur in the climacteric period, and yet not be the climacteric decline. There is a curious imitation of the Grand Climacteric that manifests itself among young women, from the age of 20 to 30 years, and which I have often observed. They appear to be fine plump healthy girls till the above period, when they begin to lose flesh, droop in spirits, grow languid and pale, with defective appetite, torpid secretions, and, in short, a general break up of the health, without any evident cause—without any tangible disease of organ or function. It is seldom fatal, though I have known it go on till death closed the scene. More frequently it takes a turn for the better—sometimes without any apparent reason—more often from some love-fit—or marriage—or TIME, which cures love-melancholy, as well as this erotic decline. Though the cause of this pseudo, or premature climacteric is not always apparent, its real nature rarely escapes the notice of the experienced physician. It is little under the control of drugs!

In several instances that have come under my own observation, and where all the symptoms of the climacteric decline—and that after the age of 60—were unequivocal, the constitution has rallied, at least for some years, and the individuals have died at last of other diseases. This has happened where especial care was taken—

> "To husband out life's taper at its close,
> And keep the flame from wasting by repose."

It was not, by *repose*, however, in the ordinary acceptation of the term—by reclining on the sofa—and stimulating a jaded appetite by provocatives. The farrago of tonics, cordials, and nutriments, in such cases, only tend to consume the pabulum of life more rapidly, and extinguish the flame more quickly. The *repose* is that of passive motion in a carriage—if possible in an open one—perpetually changing the air and scene.

The climacteric disease is not confined to a particular part, or a peculiar form. It is the breaking-up of function or structure, or both, in the weakest organ of the body. When a *function* totally or principally fails, there can be little doubt that the structure of the corresponding organ or part must be more or less changed in its molecular organization, though that change may not be visible to the eye or demonstrable by the scalpel.

---

* "Of the various immediate causes to which this malady may owe its commencement, there is none more frequent than a common cold."

"When it combines itself with a common cold, the symptoms of catarrh continue to manifest themselves, and to predominate throughout the greater part of the duration of the climacteric disease."—Sir H. HALFORD.

Although the function of digestion would seem to be the first, or amongst the first to fail, in the climacteric disease, yet it does not appear to be the one which leads directly to the final issue. Defect in assimilation (the conversion of the food into nutritious blood), is much more frequently the cause of the emaciation and debility, than the mere loss of digestive power. Dropsical effusions into the different cavities, especially those of the chest and head, are the most common forerunners of death, in the climacteric disease. The *former* occasion difficulty of breathing in ascending stairs, with some cough and wheezing:—the *latter* render the individual drowsy, stupid, forgetful, torpid, palsied—and ultimately apoplectic.

The heart, as I said before, is not unfrequently the organ on which the climacteric disease falls. It grows flabby in structure—dilated in its cavities—attenuated in its walls—and imperfect in its valves. This is the most common cause of the dropsical effusions, the difficulty of breathing, the cough—and those symptoms which, at a former period, were set down as affections of the lungs.

It would be a tedious, and, perhaps, useless task, to detail the various ways in which the CLIMACTERIC DISEASE winds up the drama of human life. The function of the KIDNEYS often fails, with corresponding change in their structure and secretion. This is a form of the climacteric disease which has been much overlooked, but which is now attracting considerable attention. The same may be said of the LIVER. Defective function in this organ prostrates the strength, and reduces the flesh, in a most extraordinary manner. It arrests nutrition, and thus subverts the powers of life, without producing any very marked phenomena that might awaken suspicion as to the cause.

It is humiliating to confess that, in climacteric diseases, palliatives only can be offered by the most skilful physician—and it is little less painful to observe the amount of mischief which is every day inflicted on humanity by rashness, empiricism, and ignorance, in such cases. Modern researches in morbid anatomy, have not enabled us to cure diseases that were previously incurable; but they have shewn us what are and what are *not* susceptible of remedy. We are thus guarded against doing harm; whilst the unprincipled charlatan, having no such check on his presumption, administers powerful drugs (for they are not remedies) in complete ignorance of the nature of the malady, and thus precipitates his victim into the grave, or, what is worse, aggravates his sufferings, during the remainder of his life!

Death, from the climacteric malady, is generally easy—and often sudden, at last. As will be shewn farther on, it is, as nearly as possible, the death of Nature, which is always easy—antedated, indeed, a few years, as to time, and considerably abridged as to duration. There is here no violent struggle between a sound constitution and an accidental illness. It is like the crumbling down, stone after stone, of an ancient castle, compared with the demolition of the same edifice, at an earlier period, by catapultæ or cannon. As the mantling ivy procrastinates the fate of the tottering tower; so, change of air and scene, with the mildest restoratives, will sometimes prolong the existence of the drooping human fabric, and add a zest to the cup of enjoyment till the bowl of life is drained!"

